# Rethinking methods used to evaluate effectiveness of therapeutics for COVID-19 and other viral respiratory illnesses

**DOI:** 10.2217/fvl-2021-0209

**Published:** 2022-01-12

**Authors:** Jean-François Rossignol

**Affiliations:** ^1^The Romark Institute for Medical Research, Tampa, FL 33607, USA

**Keywords:** antiviral drugs, clinical trial design, clinical trial endpoint, COVID-19, influenza, pandemic preparedness, respiratory infections, SARS-CoV-2, viral respiratory illnesses

There is presently a high level of interest in clinical trials of antivirals for treating viral respiratory illnesses (VRIs), specifically for COVID-19. Hundreds of trials of potential interventions are being conducted worldwide, but unfortunately, few are designed to generate reliable evidence.

Challenges in developing therapeutics for VRIs are not new. In a nonpandemic year, adults in the USA experience as many as two-to-six VRIs and children approximately six-to-eight, resulting in an estimated economic impact nearing $100 billion and thousands of hospitalizations and deaths [1–4]. Yet the few drugs approved for treating outpatient VRIs are limited to influenza, and the evidence supporting their effectiveness has been controversial.

To date, monoclonal antibodies are being used on an emergency basis for treatment of mild or moderate COVID-19 based on data indicating effectiveness in preventing hospitalizations, emergency room visits or death. However, as the proportion of the global population with SARS-CoV-2-specific immune history increases through vaccine coverage and infection, the next phase of pandemic control will focus on outcomes other than hospitalization and death.

Virologic outcomes are not considered meaningful from a clinical perspective [5,6]. Methods used for collecting and handling samples and measuring viral loads from nasopharyngeal swabs in large multicenter outpatient clinical trials have not been validated. There is also significant between-patient variability in virus strain, time from infection to initiation of treatment and host factors across clinical trial subjects, further complicating interpretability of virologic endpoints. Overcoming these issues would require strategies unreasonable for outpatient clinical trials, such as massive sample sizes or more invasive sample collection procedures [7], and even then, the clinical relevance has not been established.

Researchers are; therefore, left to assess the efficacy of antiviral therapeutics using signs and symptoms of VRIs. This also is fraught with challenges due to the self-limited nature of the illness, the variability of viruses and host factors, and the need to rely on patient-reported outcomes. Overcoming these challenges requires valid, reliable, evidence-based methods for collecting data and assessing clinically meaningful changes in patient health status. At this moment in time, there is a critical need for improvement in clinical trial design in order to improve the quality of evidence supporting effectiveness of new antiviral therapeutics for COVID-19 and other VRIs.

The challenges we now face are illustrated by clinical trials of approved influenza antiviral drugs. Symptom questionnaires used in clinical trials of the six US FDA-approved drugs for treating influenza ranged in length from 5 to 22 symptoms and were modified from one study to the next over the course of development programs. Methods of analyzing data for the primary end point also evolved independently for each drug. Ultimately, the efficacy of each approved product was assessed using a unique symptom questionnaire and primary end point in pivotal clinical trials [8–17]. The symptom questionnaires were not developed or validated in accordance with good scientific principles, and there is no evidence that the questions measured all meaningful symptoms associated with the illness, that patients understood the questions asked of them, or that the response definitions (usually some variation of the time when all symptoms are rated absent or mild and remain so for at least 21.5 h) represented a meaningful change in the patient’s health status.

Evidence from recent studies indicates that these symptom questionnaires are not comprehensive, and furthermore, several of the symptom questions represent multidimensional concepts, reducing the subject’s ability to understand the questions and impairing the ability to interpret results [18]. In this context, it is not surprising that only eight of 20 (40%) randomized controlled outpatient studies of the approved neuraminidase inhibitors (3/11 for zanamivir, 4/6 for oseltamivir and 1/3 for peramivir) demonstrated a statistically significant difference compared with the placebo in its primary analysis, and the differences measured were only about a day [13–15].

To illustrate the problem in very practical terms, the time of response in most of these influenza trials is the time at which the severity rating for the last symptom changes from moderate to mild. In most patients, the last symptom to change from moderate to mild is ‘cough’. So the change of the rating for cough from moderate to mild drives the determination of effectiveness. Yet, it is not clear that a given subject’s response to that question refers to cough frequency or severity, its impact on the subject’s ability to function or whether the cough is productive or unproductive, associated with chest tightness or difficulty breathing, etc. Furthermore, there is no evidence that a given symptom rated mild or moderate by one person is going to be rated the same by another person with the identical symptom.

Ironically, while ‘cough’ is the symptom that most often drives the response definition, it is also the symptom with the least impact on patients’ ability to function [19], which raises questions with respect to meaningfulness of the response criteria. Adding to that question, the clinical trials of influenza antivirals have frequently reported that the time of symptom alleviation (all symptoms rated absent or mild for at least 21.5 h) occurs up to a week before subjects report being at their usual state of health, and there is no apparent explanation of what happens in the interim. There is, in fact, no evidence supporting the meaningfulness of this response definition to patients, and yet, historical trials have used this data to support claims that therapeutics reduce the duration of symptoms by a day.

Inevitable consequences of using inappropriate methodology to evaluate effectiveness of therapeutics for VRIs include controversy, lack of drugs and being inadequately prepared for emerging viruses and pandemics. Reliable evidence-based methodology would allow for measuring more meaningful treatment benefit, better understanding of which patients are likely to benefit or not, higher-quality evidence and more confidence in approved medications and public health authorities.

One does not have to look far to see existing and potential controversies surrounding products offering hope in the management of outpatient COVID-19. The lack of consensus regarding COVID-19 symptoms and clinically meaningful methods for assessing treatment benefit is potentially setting the stage for a controversial or empty arsenal of drugs to mitigate the ongoing impact of the COVID-19 pandemic.

Although SARS-CoV-2 is a novel virus, historical experience with clinical trials of influenza antivirals suggests areas for improvement in pursuit of high-quality evidence from clinical trials. The point is that we need to do better. A sound evidence-based approach to the design and conduct of clinical trials is critical to the COVID-19 pandemic response and preparedness for future pandemics.

## Future perspective

At the present time, neither pharmaceutical giants nor independent investigators, regulatory bodies or other public health organizations have attempted to reach consensus on a valid, reliable methodology for assessing the effectiveness of investigational anti-SARS-CoV-2 therapeutics in outpatients based upon meaningful changes in symptoms. It is likely; therefore, that regulatory authorities will approve a few therapeutics for the treatment of COVID-19 within the next 5 years based on end points unique to each product, without clear meaningfulness and lacking the comparability necessary for clinical practice. As the pandemic wanes, development of therapeutics for outpatient VRIs will again succumb to the challenges of measuring and replicating meaningful trial results and we will again be left unprepared for the next pandemic. However, if among scientists, industry or government, a new standard is raised and valid, reliable and meaningful trial and end point designs are developed, the world may be much better prepared to respond to the pandemics of the coming decades.
